# Assessing Cognitive Ability and Simulator-Based Driving Performance in Poststroke Adults

**DOI:** 10.1155/2017/1378308

**Published:** 2017-05-07

**Authors:** Alison Blane, Hoe C. Lee, Torbjörn Falkmer, Tania Dukic Willstrand

**Affiliations:** ^1^School of Occupational Therapy and Social Work, Curtin University, Perth, WA, Australia; ^2^School of Health Sciences, Jönköping University, Jönköping, Sweden; ^3^Rehabilitation Medicine, Department of Medicine and Health Sciences (IMH), Faculty of Health Sciences, Linköping University, Pain and Rehabilitation Centre, UHL, County Council, Linköping, Sweden; ^4^School of Occupational Therapy, La Trobe University, Melbourne, VIC, Australia; ^5^Swedish National Road and Transport Research Institute (VTI), Human Factors, Göteborg, Sweden

## Abstract

Driving is an important activity of daily living, which is increasingly relied upon as the population ages. It has been well-established that cognitive processes decline following a stroke and these processes may influence driving performance. There is much debate on the use of off-road neurological assessments and driving simulators as tools to predict driving performance; however, the majority of research uses unlicensed poststroke drivers, making the comparability of poststroke adults to that of a control group difficult. It stands to reason that in order to determine whether simulators and cognitive assessments can accurately assess driving performance, the baseline should be set by licenced drivers. Therefore, the aim of this study was to assess differences in cognitive ability and driving simulator performance in licensed community-dwelling poststroke drivers and controls. Two groups of licensed drivers (37 poststroke and 43 controls) were assessed using several cognitive tasks and using a driving simulator. The poststroke adults exhibited poorer cognitive ability; however, there were no differences in simulator performance between groups except that the poststroke drivers demonstrated less variability in driver headway. The application of these results as a prescreening toolbox for poststroke drivers is discussed.

## 1. Introduction

It is well-established that safe driving, as an important activity for daily living, is heavily reliant on functioning cognitive processes [1, 2]. It is also well acknowledged that cognitive processes decline following a stroke and that this may impact on their ability to drive [3]. There is great debate regarding whether poststroke adults are at an increased risk of a crash, with much variation in the results. For example, some research has suggested that poststroke drivers are up to three times more likely to crash [4], whereas others have suggested there is no association with increased crash risk [5]. With this uncertainty and the knowledge that this population will only increase [6], knowing the extent of cognitive decline and the safe limit required in order to return to driving on the road is essential.

It has been estimated that between 30% and 50% of poststroke adults will return to driving [7, 8]. Currently, the Australian process for returning to driving after a stroke (using a private vehicle license) requires the affected person to wait a minimum of two weeks for a transient ischemic attack and four weeks for a fully ischemic stroke or haemorrhagic stroke [9]. All poststroke adults must also inform the relevant state transport-licensing authority of their stroke and obtain medical clearance prior to driving [9]. Only a medical professional (usually a general practitioner) can advise whether a person is safe to drive, and if the respective medical professional believes it is necessary, the poststroke adult may be required to attend a driving assessment or further driver training before returning to the road [9]. Ideally, poststroke adults with questionable driving capability should undertake a two-stage assessment process that involves a neurological examination and an on-road observation; however, limited guidelines are available to help doctors determine fitness-to-drive [10]. Although on-road driving tests remain the “gold standard” for driving assessments, they are arguably subjective, highly stressful, costly, and time consuming [11, 12], as well as carry inherent risks to safety [13]. Therefore, using off-road techniques to give an accurate estimate of the stage of recovery, as well as provide reliable information for targeted driver rehabilitation, is required. Neuropsychological assessments have regularly been implemented to assess cognition, and there is a strong correlation between the results and the client's driving performance; however, cognitive ability should be assessed alongside actual driving behaviour, rather than in isolation [3].

Driving simulation is one of the most heavily utilised alternative measures of driving performance [14]. This is due to its ability to reliably measure driving performance, whilst also ensuring driver safety and eliminating difficult to control extraneous variables, such as traffic density and weather conditions [11, 14]. The limited research using driving simulators to assess driving performance in a poststroke population has found varying results. Some studies have reported that poststroke adults performed significantly worse in a driving simulator assessment, having exhibited more errors when compared to controls [15–17]. Others have reported that although poststroke drivers exhibit difficulty with secondary tasks, such as listening span tasks whilst driving, there were no differences in actual driving performance variables [18]. Part of the reason for the discrepancy and subsequent predictive value of previous simulator-based research may be due to the fact that the majority of simulated driving research has focused on unlicensed poststroke drivers. It stands to reason that in order to determine whether simulators and cognitive assessments can accurately assess driving performance, the baseline should be set by those who are already licensed to drive. Therefore, the aim of this study was to assess differences in driving simulator performance in licensed community-dwelling poststroke drivers and controls, as well as to assess whether differences in cognition account for differences in driving performance.

## 2. Method

### 2.1. Design

A quasi-experimental comparison group design, involving a group of licensed poststroke drivers and a control group of licensed drivers of similar ages and of the same gender, was utilised to perform the assessments.

### 2.2. Participants

The inclusion criteria for study participation were that all participants held a driving license valid within Australia, had at least one year of overall driving experience, drove at least twice a week, and had access to a fully insured vehicle. Further criteria for the poststroke group were that they had previously been diagnosed with a stroke (either ischemic or haemorrhagic) and had obtained medical clearance to drive. Poststroke participants self-reported their condition; however, where possible, participants were asked to bring in any medical documents relating to their stroke, for demographic verification purposes. Participants were excluded if they had been diagnosed with any neurodegenerative disease, such as Parkinson's disease or dementia, or if they required a wheelchair for mobility. Some poststroke participants had hemiparesis and used assistive equipment whilst driving (e.g., a steering knob, modified pedals, or foot brace); however, as all participants were community dwelling and were driving their own vehicles, they were considered a level two or below on the modified Rankin Scale [19, 20]. Participants were recruited using purposive sampling techniques, including speaking at and recruiting from local community groups and poststroke support groups, as well as advertising in community newspapers and on local radio stations. The recruitment and assessment of participants took place between April 2015 and February 2016.

The total sample consisted of 80 participants including 37 poststroke drivers (male = 30) and 43 controls (male = 35). The mean age of the poststroke group was 65 years (SD = 9) and the ages ranged from 37–81 years. The mean age of the control group was slightly older at 66 years (SD = 7), and ages ranged from 49–81 years; however, the age difference between groups was not significant, *t* = −0.61, df = 81, and *p* < 0.05. Driving exposure data were collected from each participant as an estimate of their annual millage in kilometres (km). The poststroke group reported a mean of 15,529 km (SD = 12,440), whereas the controls reported 15,341 km per year (SD = 8066) and the difference was nonsignificant *t* = 0.78, df = 70, and *p* < 0.05. The license length of the poststroke group was slightly shorter than that of the control group with means of 47 years, (SD = 8) and 49 years (SD = 8), respectively, with the difference again nonsignificant, *t* = −0.86, df = 78, and *p* < 0.05. As there were no significant differences found for these variables, based on previously researched driving criteria, the participants were considered well matched at group level [21].

### 2.3. Measures

#### 2.3.1. Driving Simulator

**Figure 1:**
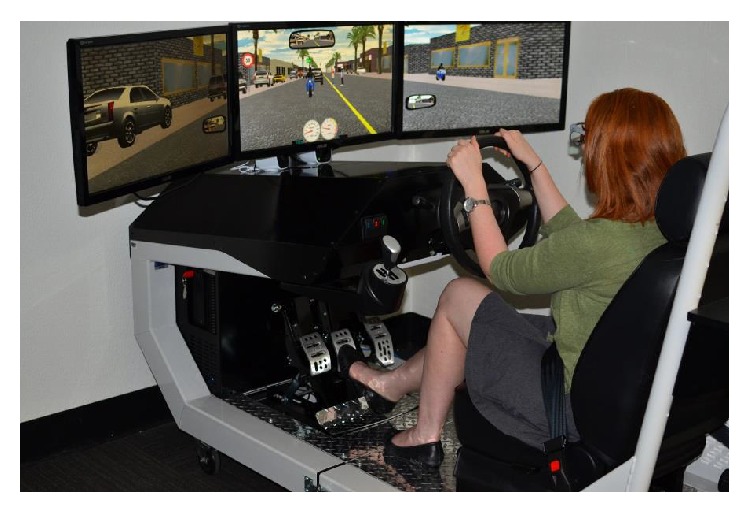
The Curtin University STISIM driving simulator.

The driving simulator based at the Curtin University School of Occupational Therapy and Social Work is a fixed-base car driving simulator that has midlevel physical fidelity and consists of a driving console with three ASUS (24″ 16 : 9 ratio) display screens, onto which the forward facing and peripheral driving scenes are displayed (Figure 1()). For consistency, the simulator was configured to use automatic transmission for all participants. A steering knob was installed onto the steering wheel if required by the participant, and the acceleration pedal could be configured as either the left side pedal or right side pedal. This was implemented in order to best simulate any vehicle modifications found in the participant's own vehicle.

Driving scenarios were programmed using the STISIM^©^ driving software [22]. A practice scenario that lasted approximately 10 minutes and included a 60 km per hour dual-lane road was utilised for each participant, in order to familiarise them with the controls and visual stimuli. The experimental scenarios consisted of a lead-car scenario and an emergency stop scenario, which both contained 60 km per hour roads along a two-lane suburban road and a four-lane suburban road, respectively. The lead-car scenario required participants to follow a lead-car for the duration of the scenario, and their main task was to maintain a safe, consistent distance between themselves and the car in front. The emergency stop scenario required participants to apply the brake as soon as a stop sign appeared and their time to react was recorded. Due to the limited time availability of enrolled participants (5 participants including 4 controls and 1 poststroke driver) or the onset of simulator sickness (22 participants including 11 controls and 11 poststroke drivers), 27 participants did not complete the emergency stop scenario. A further 5 participants (3 controls and 2 poststroke drivers) failed to stop within the allotted time for the emergency stop task; therefore, no reaction time data were recorded for these participants and the total number of responses recorded for the emergency stop scenario was 48.

#### 2.3.2. Cognitive Measures

A series of cognitive measures were utilised to assess different aspects of cognition: psychomotor processing, attention, executive function, propensity for risk taking, spatial cognition, and visuospatial function.

*(1) Psychomotor Processing.* Following criteria and procedures previously implemented in similar cognitive research [23, 24], the participants completed a simple reaction time task (SRT), a two-choice reaction time task (2CRT), and a four-choice reaction time task (4CRT). On all tasks, the participants were instructed to answer both as quickly and as accurately as possible. Hit, miss, and false alarms (where appropriate) were recorded. In order to account for response bias, all response data reported were adjusted for hit rate. Baseline reaction time was measured using the SRT. Participants were presented with the letter X in the middle of the display and told to press the space bar as soon as the X appeared. For the 2CRT and 4CRT, participants were instructed to press the specified key corresponding to where the circle spatially appeared on the screen. All targets were presented randomly. A higher rate of accuracy and lower reaction times were indicators of increased performance.

*(2) Attention and Executive Function.* There were two types of attention measured as part of this study: selective attention and divided attention. Both were measured using the Useful Field of View^©^ (UFOV) task. The Useful Field of View task was developed as a means of measuring visual, selective, and divided attentions [25] and has been regularly implemented in poststroke driving research [26, 27]. Shorter processing speeds for accurate answers were indicative of greater performance.

A second selective attention task, utilised in previous driving research [24], was administered to assess raw individual reaction times to visual search stimuli. Using E-Prime 2.0 [28], participants were presented with a 6 × 6 rectangular arrangement of multiple letter O's and told to look for the embedded target letter (Q) amongst them. The instructions were, after each display, to press the corresponding key (X for yes, M for no) on the keyboard to determine the presence of a Q as quickly and accurately as possible. Throughout the 64 experimental trials, the number of yes and no answers was equally distributed; however, the sequence was randomised for each participant. Shorter reaction times for accurate answers were indicative of greater performance.

Executive function was assessed using the Delis-Kaplan Trail Making Task^©^ (DKEFS; [29]). The task included 5 individual component tasks that assessed different levels of cognition. Task 1 was a visual cancellation task and tasks 2, 3, and 5 comprised several connect the circle tasks, which were used to provide a baseline performance score of key components of cognition used within executive function, specifically, visual scanning, number sequencing, letter sequencing, and motor speed, respectively [30]. Task 4 was a number-letter switching task, which was used to asses executive function, specifically through assessing visuospatial thought flexibility [30]. Each task was paper-based and participants were timed to completion. The total time taken for each task was recorded and in order to control for the baseline functions represented in trail tasks 1–3, and 5, contrast scores were calculated [29]. The raw time-to-completion scores were scaled in order to account for age effects [29]. Higher scaled scores were indicative of greater performance. The tests have previously been used to assess cognitive performance in poststroke adults and found to be sensitive to cognitive decline, particularly in frontal lobe function [31, 32].

*(3) Propensity for Risk.* The Balloon Analogue Risk Task (BART; [33]) was used to measure propensity for risk-taking and aversive behaviour. The objective of the task was to pump up the balloon displayed on screen and collect the most points without allowing the balloon to burst. The number of pumps required for the balloon to burst was randomised and ranged between 1 and 128 pumps, which is the standard for the BART [33]. Participants with lower scores and fewer burst balloons were considered to be risk aversive, whereas participants with higher scores and more burst balloons had a greater propensity for risk. The BART was administered using E-Prime v 2.0 Software and the number of balloons saved, as well as the adjusted average number of pumps for each balloon recorded.

*(4) Spatial Cognition and Visuospatial Function.* Poststroke adults often experience deficits in visuospatial function [34, 35]; therefore, to test this, the Block Design task (a subtask of the Wechsler Adult Intelligence Scale) was used to measure nonverbal visuospatial reasoning [36]. The test involved constructing a specified design made out of a series of cuboid blocks as quickly and as accurately as possible. The design increased in difficulty with each alteration and the time limit allowed was extended to correspond with the increased difficulty. Participants were scored depending on the number of designs completed within each allocated time. A time bonus was added depending on the time taken to complete each design. The task was discontinued once the participant had failed to complete two consecutive designs within time. Higher scores were indicative of greater performance.

Spatial cognition, perception, and orientation ability were measured using the Benton Judgement of Line Orientation task [37]. The test consisted of a series of reference lines arranged in a semicircle, from which participants had to identify specified target lines. The rotation and angle alteration of the target lines increased the difficulty of the task as the test progressed. The amount of correctly identified lines was recorded. Higher scores were indicative of greater performance.

### 2.4. Data Collection and Procedure

Participants were provided with a participant information sheet and screened for minimum visual acuity using the revised charts 1–3 from the 2000 ETDRS visual acuity chart [38]. All participants displayed visual acuity greater than or equal to 20/40, which is the minimum level to legally drive in Western Australia. This screening was implemented to control for any confounding influence of poor visual acuity in the cognitive and driving simulation scenarios. Participants completed the cognitive tasks before completing the tasks in the simulator, and the order of completion for the cognitive tasks was randomised for each participant using the Latin square method [39]. Overall, the process lasted approximately 1½ hours.

### 2.5. Data Analysis and Processing

Performance in the simulator was assessed by calculating the moment-to-moment instability of each driving-dependent variable (headway, speed, lateral lane position, and steering input), with the measures of central tendency used to report braking reaction time. Finally, the cumulative number of speed exceedances and the cumulative number of crashes in the follow-car scenario were calculated. Reaction time in the simulator was assessed using two variables: braking reaction time and braking stopping time. Braking reaction time was defined as the time it took for the participant to react to the stop sign, and the braking stopping time was the total amount of time taken for the car to come to a complete stop following the stop sign. Following previously implemented driving simulator research [24], analysis consisted of performing a standard error regression with each driving variable (headway, speed, lateral lane position, and steering input), listed as the dependent variable and the time recorded in 0.5 second increments as the independent variable. This technique was used to provide a collated moment-to-moment measure of instability consisting of residuals for the target driving variable [40]. The process of calculating the residual data to be used as a dependent variable in the final analysis was conducted using a Microsoft Excel-based macro, programmed using Visual Basic for Applications [41].

### 2.6. Statistical Analysis

Normality of all variables was assessed using a histogram, box-plots, and Q-Q plots, as well as measures of skewness and kurtosis. A Box-Cox transformation was conducted on the UFOV data, DKEFS data, visual search data, reaction time task data, and headway calculation to improve normality. Between-group differences in simulator performance and cognitive performance were assessed using independent sample *t*-tests. Following transformation, analysis of non-normally distributed data between groups was performed using a Mann–Whitney *U* test.

For the cognitive variables and driving simulator performance variables, with 37 and 43 participants in the respective groups, there was 80% power to detect an effect size of 0.64 between the poststroke drivers and controls [42]. For the braking reaction time task, due to significant drop out, there were 22 participants in the smallest group and the standardised difference value dropped. Therefore, there was 80% power to detect an effect size of 0.82. A *p* value of <0.05 was taken to indicate a statistically significant association in all tests.

### 2.7. Ethical Considerations

This research and the associated study protocols were approved by the Curtin University Human Research Ethics Committee—approval number HR206-2014 and conformed to the principles of the Declaration of Helsinki. Participants were presented with an information sheet and given the opportunity to ask questions, and each provided signed informed consent prior to participation. Participants were also informed that they could leave the study at any time without incurring any negative consequences. Participants were offered a gift voucher worth $15 (Australian) as compensation for their time and participation.

## 3. Results

### 3.1. Differences in Simulator

**Figure 2:**
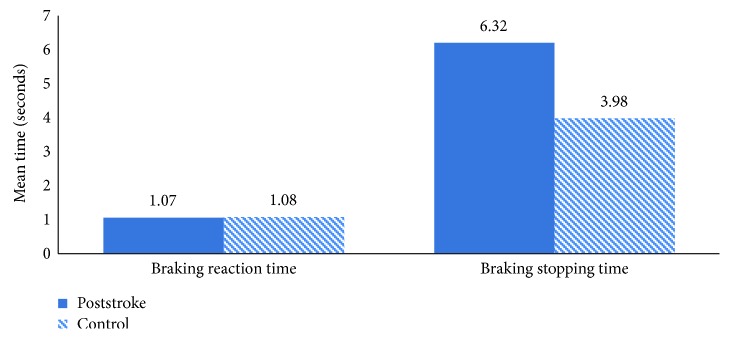
The mean braking reaction time and stopping time in seconds for the poststroke and control groups.

Overall, the poststroke drivers displayed greater variability in speed, lateral lane position, and steering input, suggesting that the poststroke drivers varied their speed more often, varied their position on the road, and moved the steering wheel more than the control group; however, these results were nonsignificant at group level (Table 1). Interestingly, although the poststroke drivers were on average, quicker to respond to the braking task, they took longer to fully stop the car (Figure 2()); although again, this result was nonsignificant at group level (Table 1). The main significant difference between the groups was that the poststroke group displayed less variability in headway (Table 1) suggesting that the poststroke drivers' headway was more consistent than the control group.

**Figure 3:**
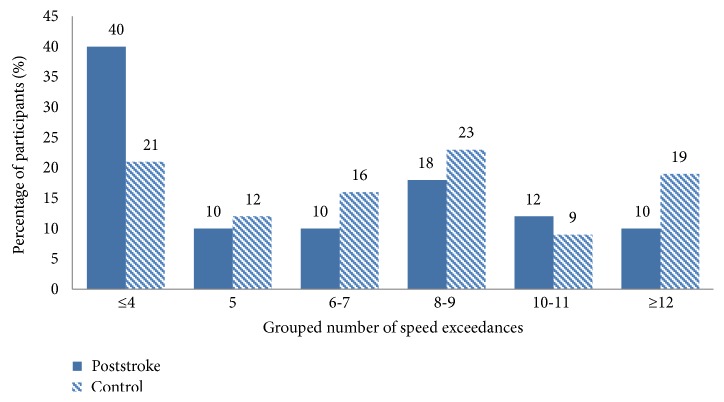
The grouped number of speed exceedances in the poststroke and control groups.

There was also no between-groups difference reported for cumulative number of crashes, as there were zero crashes reported. The speed exceedances were grouped into 6 proportionally distributed categories using the binning function in SPSS. The number of speed exceedances recorded was relatively evenly distributed across both groups although the largest differences appeared to be that 40% of poststroke drivers were recorded as having committed ≤4 speed exceedances compared to 21% of the control group, and 10% of the poststroke drivers recorded ≥12 speed exceedances compared to 19% of the control groups (Figure 3()). However, overall, there was no significant difference in speed exceedances between groups *X*^2^ = 4.76, df = 5, and *p* > 0.05.

### 3.2. Differences in Cognition

There were significant between-groups differences in several of the cognitive variables: BART, visual search, D-KEFS—Number Letter Switching, and UFOV (Tables 2 and 3). The poststroke drivers saved more balloons in the BART and also had a lower average amount of pumps per balloon, both of which indicate a greater tendency for risk averseness than those in the control group. In the UFOV divided attention task, UFOV selective attention task, and visual search tasks, the poststroke drivers had significantly longer reaction times, all of which indicated poor performance. There were no statistically significant differences between groups for any of the following measures: BJLOT, block design, UFOV 1, or any of the psychomotor tasks.

## 4. Discussion

### 4.1. Summary of Findings

As mentioned in the introduction, full driving assessments, i.e., including an on-road component, are both time consuming and costly [11, 12] and constitute a real risk to both the candidate and the assessor [13]. Furthermore, they inherently comprise an uncontrollable component as other road users' actions and interactions cannot be controlled [14, 43]. Hence, if there was a safe way to assess poststroke drivers prior to returning to drive, it would not only save time and money but it would actually increase road safety. The only problem with this is knowing what to assess and how to make those assessments relevant to the fact that potential poststroke drivers will predominantly be older. In the present study, a combination of driving simulator scenarios and psychometric tests was implemented in order to assist with that decision; the idea being that an actual poststroke driving profile would provide support for determining where they would differ from the nonaffected older driver.

Whilst most simulator driving variables did not differ between the two groups, as was expected, given that all participants were licensed drivers, of greater concern was the finding that the older adults in the control group varied their headway more than the poststroke adults. This was an unexpected finding, albeit consistent with the simultaneous finding that on-average, poststroke drivers were more risk aversive than controls. Headway is a known predictor of safe driving and has been established as a valuable assessment in simulator research [44]; therefore, this finding is also quite challenging given that the present study aimed to identify poststroke-specific indicators that could assist driving assessments by allied health professionals. The fact that the control group performed better in several of the cognitive tasks yet displayed more variable headway appears counterintuitive; however, when taken with the result that the poststroke drivers were more risk aversive, this may suggest that the less headway variation in the poststroke group is due to the licensed drivers' awareness of their limitations and amending their driving as a result of executive function deficits. Although further exploration of this relationship is beyond the scope of the present study, more research is planned to investigate this, specifically, whether poststroke awareness of cognitive deficits is associated with amended driving behaviour.

In many of the cognitive tests, the poststroke drivers were found to have poorer scores than their control group counterparts. These findings corroborate previous research, suggesting that a mixture of off-road neuropsychological tasks, as well as using a driving simulator, may be beneficial in establishing who is safe to drive, prior to undertaking an on-road assessment [45]. Specifically, this study suggests that a battery of tasks assessing attention, baseline cognition, executive functions, and propensity for risk (i.e., the UFOV, the D-KEFS TMT, and the BART) may be a useful screening tool for poststroke drivers, prior to undertaking a full on-road driving assessment. Furthermore, these tasks may also provide a baseline for assistive poststroke driver training. The final finding of the current study is that the following tests did not contribute to the pre-on-road screening; psychomotor processing ability (SRT, 2CRT, and 4CRT); spatial cognition, perception, and orientation ability (BJLOT); and visuospatial function (Block Design). This suggests that although poststroke drivers may be lacking in these cognitive skills, ultimately, these deficits may not affect their ability to drive and therefore using the respective assessments as a prescreening for driving ability is unnecessary.

### 4.2. Limitations

As with all simulator research, there is much debate on the use of driving simulators for driving assessments [14]. This study aimed to emulate driving on-road in a safe, replicable environment for the purpose of establishing baseline driving performance in licensed drivers; therefore, a simulator was used, which has previously been validated for on-road driving performance [46]. Similarly, although on-road driving performance was not the focus of the study, the participants sampled were those who were licensed drivers. This was in order to ensure that all participants were of a sufficient standard to drive, facilitating generalisation to on-road driving capability. Despite the driving simulator being validated, it should also be noted that participants spent a relatively short amount of time in the driving simulator, which can limit the representativeness of the results [43]. This was partly due to the measurements being assessed and partly to minimise the effects of simulator sickness [43]. As part of this research, the majority of participants attempted all scenarios; however, there was a relatively small attrition rate for the emergency stop task as many of the participants developed simulator sickness and the assessment was halted. There was also a small number of participants who did not have data recorded for some cognitive assessments, due to a mixture of logging errors, participant choice, or restraints on the participant's time. Due to this attrition rate and the initial relatively small sample size, there were fewer participants with recorded data, which increases the potential for a type II error; therefore, the results should be interpreted with caution and further research with a larger sample is required for verification.

## 5. Conclusion

Although poststroke adults have decreased cognitive ability, they can perform at a similar level in a driving simulator to licensed controls. As this research was conducted on licensed drivers, it presents a baseline indication for the use of off-road and simulator assessments as predictive tools. Specifically, the study suggests that driving simulators and those tasks assessing performance in propensity for risk, executive function, selective attention, and divided attention may be useful for future researchers and clinicians as assessment, rehabilitation, and training tools for nonlicensed poststroke adults who wish to return to driving.

## Figures and Tables

**Table 1:** The between-group differences in simulator performance.

Variables	Poststroke driver			Controls			*t* value	df	*p* value
	*n*	Mean	SD	*n*	Mean	SD			
Headway	37	3.26	1.87	43	1.71	1.36	−2.20	48.58	0.03^∗^
Lateral lane position	37	0.73	0.39	43	0.64	0.35	1.18	78	0.24
Speed	37	8.83	1.75	43	8.41	1.14	1.29	78	0.20
Steering input	37	1.48	1.06	43	1.13	0.66	1.80	78	0.13
Braking reaction time	22	1.07	0.31	26	1.08	0.27	−0.19	46	0.85
Braking stopping time	22	6.32	11.03	26	3.98	1.35	1.07	46	0.29

^∗^ *p* value < 0.05.

**Table 2:** The between-group analysis of significant cognitive variables.

Variables	Poststroke driver			Controls			*t* value	df	*p* value
	*n*	Mean	SD	*n*	Mean	SD			
BART average number of pumps per balloon	37	25.16	3.32	43	27.49	17.78	2.46	78	0.02^∗^
BART average saved balloons	37	20.15	10.18	43	22.90	4.71	−2.31	68.51	0.02^∗^
BJLOT	36	24.64	5.07	43	25.44	9.93	−0.78	65.33	0.44
Block design	37	37.60	10.50	43	40.40	10.22	−1.21	78	0.23
SRT (milliseconds)	34	375.59	161.35	43	244.19	78.05	1.12	78	0.27
2CRT (milliseconds)	34	97.99	172.91	43	50.58	88.64	1.46	46.56	0.15
4CRT (milliseconds)	34	427.62	258.67	43	333.33	255.92	1.60	75	0.11
Visual attention (simple visual search task)	34	693.99	422.08	43	494.11	150.97	2.89	39.70	0.01^∗^
UFOV divided attention	35	82.89	112.33	42	36.29	38.84	2.52	40.78	0.02^∗^
UFOV selective attention	35	144.31	110.33	41	90.13	50.70	2.82	46.09	0.01^∗^

	*n*	Median	Mean	IQR	*n*	Median	Mean	IQR	*p* value
UFOV processing speed	35	13.8	19.31	0.10	42	13.8	14.53	0.10	0.82

^∗^Significant data; *p* value < 0.05.

**Table 3:** The between-group analysis of D-KEFS TMT variables.

Variables	Poststroke driver			Controls			*t* value	df	*p* value
	*n*	Mean	SD	*n*	Mean	SD			
Visual scanning (D-KEFS TMT 1)	37	7.38	3.62	43	10.90	2.17	−5.19	57.06	0.001^∗∗^
Numerical sequencing and visual attention (D-KEFS TMT 2)	37	9.08	3.75	43	11.47	3.01	−3.16	78	0.003^∗^
Letter sequencing, visual attention, and visuomotor abilities (D-KEFS TMT 3)	37	8.51	4.07	43	11.72	2.57	−4.14	58.97	0.001^∗∗^
Cognitive flexibility and executive function (D-KEFS TMT 4)	37	8.27	4.43	43	11.65	2.35	−4.16	52.86	0.001^∗∗^
Motor and processing speed (D-KEFS TMT 5)	37	9.68	2.92	43	12.02	1.71	−4.30	56.26	0.001^∗∗^
Combined number sequencing and letter sequencing (D-KEFS TMT 2 + TMT 3)	37	9.12	4.02	43	12.35	2.89	−4.04	64.29	0.001^∗∗^

	*n*	Median	Mean	IQR	*n*	Median	Mean	IQR	*p* value
D-KEFS cognitive flexibility and executive function contrast score (D-KEFS TMT 4–2 & 3)	37	1.00	2.92	4.50	43	1.00	1.67	0.00	0.02^∗^

^∗^ *p* value < 0.05. ^∗∗^*p* value < 0.001.

## References

[1] Anstey K. J., Wood J., Lord S., Walker J. G. (2005). Cognitive, sensory and physical factors enabling driving safety in older adults. *Clinical Psychology Review*.

[2] Groeger J. A. (2000). *Understanding Driving: Applying Cognitive Psychology to a Complex Everyday Task*.

[3] Marshall S. C., Molnar F., Man-Son-Hing M. (2007). Predictors of driving ability following stroke: a systematic review. *Topics in Stroke Rehabilitation*.

[4] Perrier M.-J., Korner-Bitensky N., Petzold A., Mayo N. (2010). The risk of motor vehicle crashes and traffic citations post stroke: a structured review. *Topics in Stroke Rehabilitation*.

[5] Haselkorn J. K., Mueller B. A., Rivara F. A. (1998). Characteristics of drivers and driving record after traumatic and nontraumatic brain injury. *Archives of Physical Medicine and Rehabilitation*.

[6] Strong K., Mathers C., Bonita R. (2007). Preventing stroke: saving lives around the world. *The Lancet Neurology*.

[7] Fisk G. D., Owsley C., Pulley L. V. (1997). Driving after stroke: driving exposure, advice, and evaluations. *Archives of Physical Medicine and Rehabilitation*.

[8] Bryer R. C., Rapport L. J., Hanks R. A. (2006). Chapter 7 - determining fitness to drive: neuropsychological and psychological considerations A2 - Pellerito, Joseph Michael. *Driver Rehabilitation and Community Mobility*.

[9] Austroads and National Transport Commision Australia (2016). *Assessing Fitness to Drive for Commercial and Private Vehicle Drivers: Medical Standards for Licensing and Clinical Management Guidelines*.

[10] Murie-Fernandez M., Iturralde S., Cenoz M., Casado M., Teasell R. (2014). Driving ability after a stroke: evaluation and recovery. *Neurología (English Edition)*.

[11] Lee H. C., Cameron D., Lee A. H. (2003). Assessing the driving performance of older adult drivers: on-road versus simulated driving. *Accident Analysis & Prevention*.

[12] Lee H. C., Lee A. H. (2005). Identifying older drivers at risk of traffic violations by using a driving simulator: a 3-year longitudinal study. *The American Journal of Occupational Therapy*.

[13] Galski T., Ehle H. T., MCdonald M. A., Mackevich J. (2000). Evaluating fitness-to-drive after cerebral injury: basic issues and recommendations for medical and legal communities. *The Journal of Head Trauma Rehabilitation*.

[14] Stedmon A., Young M., Hasseldine B. Keeping it real or faking it: the trials and tribulations of real road studies and simulators in transport research.

[15] Hird M. A., Vesely K. A., Christie L. E. (2015). Is it safe to drive after acute mild stroke? A preliminary report. *Journal of the Neurological Sciences*.

[16] McKay C., Rapport L. J., Bryer R. C., Casey J. (2011). Self-evaluation of driving simulator performance after stroke. *Topics in Stroke Rehabilitation*.

[17] Kotterba S., Widdig W., Brylak S., Orth M. (2005). Driving after cerebral ischemia—a driving simulator investigation. *Wiener Medizinische Wochenschrift*.

[18] Lundqvist A., Gerdle B., Ronnberg J. (2000). Neuropsychological aspects of driving after a stroke—in the simulator and on the road. *Applied Cognitive Psychology*.

[19] Rankin J. (1957). Cerebral vascular accidents in patients over the age of 60: II. Prognosis. *Scottish Medical Journal*.

[20] van Swieten J. C., Koudstaal P. J., Visser M. C., Schouten H. J., van Gijn J. (1988). Interobserver agreement for the assessment of handicap in stroke patients. *Stroke*.

[21] Evans L. (2004). *Traffic Safety*.

[22] Allen R. W., Stein C. A., Aponso B. L. A low cost, part task driving simulator based on microcomputer technology.

[23] Bunce D., Handley R., Gaines S. O. (2008). Depression, anxiety, and within-person variability in adults aged 18 to 85 years. *Psychology and Aging*.

[24] Bunce D., Young M. S., Blane A., Khugputh P. (2012). Age and inconsistency in driving performance. *Accident Analysis & Prevention*.

[25] Ball K., Owsley C. (1993). The Useful Field of View test: a new technique for evaluating age-related declines in visual function. *Journal of the American Optometric Association*.

[26] Fisk G. D., Owsley C., Mennemeier M. (2002). Vision, attention, and self-reported driving behaviors in community-dwelling stroke survivors. *Archives of Physical Medicine and Rehabilitation*.

[27] George S., Crotty M. (2010). Establishing criterion validity of the Useful Field of View assessment and stroke drivers’ screening assessment: comparison to the result of on-road assessment. *American Journal of Occupational Therapy*.

[28] Schneider W., Eschman A., Zuccolotto A. (2012). *E-Prime 2.0*.

[29] Delis D. C., Kaplan E., Kramer J. H. (2001). *Delis-Kaplan Executive Function System (D-KEFS) Examiner’s Manual*.

[30] Swanson J. (2005). The Delis-Kaplan Executive Function System: a review. *Canadian Journal of School Psychology*.

[31] Homack S., Lee D., Riccio C. A. (2005). Test review: Delis-Kaplan Executive Function System. *Journal of Clinical and Experimental Neuropsychology*.

[32] Wolf T. J., Rognstad M. C. (2013). Changes in cognition following mild stroke. *Neuropsychological Rehabilitation*.

[33] Lejuez C., Read J. P., Kahler C. W. (2002). Evaluation of a behavioral measure of risk taking: the Balloon Analogue Risk Task (BART). *Journal of Experimental Psychology: Applied*.

[34] Kaplan J., Hier D. B. (1982). Visuospatial deficits after right hemisphere stroke. *American Journal of Occupational Therapy*.

[35] Stone S., Wilson B., Wroot A. (1991). The assessment of visuo-spatial neglect after acute stroke. *Journal of Neurology, Neurosurgery & Psychiatry*.

[36] Wechsler D. (2008). *Wechsler Adult Intelligence Scale–Fourth Edition (WAIS–IV)*.

[37] Benton A. L., Hamsher K. D., Varney N. R., Spreen O. (1983). *Judgment of Line Orientation*.

[38] Precision Vision (2000). *Series Revised ETDRS Charts*.

[39] Grant D. A. (1948). The Latin square principle in the design and analysis of psychological experiments. *Psychological Bulletin*.

[40] Bloomfield J. R., Carroll S. A., Robertson S. A. (1996). New measures of driving performance. *Contemporary Ergonomics*.

[41] Microsoft Office (2010). *Visual Basic for Applications*.

[42] Cohen J. (1988). *Statistical Power Analysis for the Behavioral Sciences*.

[43] Carsten O., Jamson A. H., Porter B. E. C. (2011). Driving simulators as research tools in traffic psychology. *Handbook of Traffic Psychology*.

[44] Vogel K. (2003). A comparison of headway and time to collision as safety indicators. *Accident Analysis & Prevention*.

[45] Lew H. L., Poole J. H., Lee E. H., Jaffe D. L., Huang H. C., Brodd E. (2005). Predictive validity of driving-simulator assessments following traumatic brain injury: a preliminary study. *Brain Injury*.

[46] Lee H. C., Lee A. H., Cameron D. (2003). Validation of a driving simulator by measuring the visual attention skill of older adult drivers. *The American Journal of Occupational Therapy*.

